# Nerve wrap after end-to-end and tension-free neurorrhaphy attenuates neuropathic pain: A prospective study based on cohorts of digit replantation

**DOI:** 10.1038/s41598-017-19134-8

**Published:** 2018-01-12

**Authors:** Xiaozhong Zhu, Haifeng Wei, Hongyi Zhu

**Affiliations:** Department of Orthopaedic Surgery, Shanghai Jiaotong University Affiliated Sixth People’s Hospital, Shanghai, 200233 China

## Abstract

The repair of injured peripheral nerve is still challenging for surgeons. The end-to-end and tension-free neurorrhaphy is the current gold standard for reconstruction after complete nerve transection without significant defect. The main objective of this study neurorrhaphy in digit replantation affects the sensory recovery and neuropathic pain in replanted digit. Total 101 patients who received replantation of single completely amputated digit were included for analysis in this study. In group I (n = 49), the digital nerves were repaired with end-to-end and tension-free neurorrhaphy and then wrapped into a tendon-derived collagen nerve conduit. In group II (n = 52), the digital nerves were repaired with end-to-end and tension-free neurorrhaphy only. The static two-point discrimination (s2PD) was performed to evaluate sensory recovery. Visual analog scale (VAS) scores of pain at rest and with exertion were measured respectively. The s2PD tests at three and six months after surgery did not show any significant difference between the two groups. The VAS scores at rest and with exertion of group I were significantly reduced compared with those of group II at three and six months after surgery. Thus, we concluded that nerve wrap into a collagen conduit after end-to-end and tension-free neurorrhaphy could attenuate neuropathic pain after digit replantation but have no benefit for sensory recovery.

## Introduction

The repair of vessels, nerves, tendons and bones of amputated digit is essential for the survival and functional recovery of replanted digit^1–3^. After the advance of microsurgery in decades, the survival rates of digital replantation have been reported up to 80%^4,5^. A satisfactory range of motion and appearance could also be restored by primary replantation and secondary surgeries, if necessary, in most cases^6^. However, the repair of injured nerve is still challenging in digit replantation. The recovery after nerve injury is commonly incomplete and disappointing even when the injured nerves were repaired without any tension by microsurgical techniques^7^. In addition, neuropathic pain can develop after nerve injury, when deleterious changes occur in injured nerve^8^. A previous study has revealed that the neuropathic pain can occur in up to 45% patients after replantation^9^.

The end-to-end and tension-free neurorrhaphy is the current gold standard for reconstruction after complete nerve transection without significant defect. Nerve wrap with biodegradable conduits, also known as nerve tubulization, is widely applied as a strategy in treatment of small nerve defect^10^ and multiple studies has demonstrated that nerve wrap has superior or similar outcomes compared with tensile neurorrhaphy (neurorrhaphy with tension) and nerve graft^10–12^.

One important mechanism that influences recovery and causes neuropathic pain is the scar formation at the site of nerve anastomosis^8,13–15^. Recent progress based on the nerve transection model in rats has demonstrated that nerve wrap after end-to-end and tension-free neurorrhaphy could decrease perineural scar tissue formation^16,17^. However, there is few literature, to the best of our knowledge, evaluating the clinical efficacy of nerve wrap after end-to-end and tension-free neurorrhaphy.

## Methods

### Ethical approval

The study was approved by the Ethics Committee of Shanghai Jiaotong University Affiliated Sixth People’s Hospital. Informed consent was obtained from all donors in accordance with the Declaration of Helsinki.

### Participants and study setting

Total 101 patients who received replantation of single completely amputated digit from 1 March 2016 to 1 March 2017 were included in this study. The Tamai’s level of amputation was classified according to the classification system described previously^18^. For cases amputated at Tamai level I and II, there might be no nerve suitable for repair. Hence, this study only included patients with amputation at Tamai level III, IV and V. Complete amputations were defined as complete separation of all parts without any bridging tissues. Patients with any of the following criteria were excluded: nerve defect, single or multiple organ failure, peripheral arterial diseases, additional wound on artery in the ipsilateral arm or forearm, age below 18, replant failure, peripheral neuropathy before injury. Patient characteristics including age, smoking, amputation level, and mechanism of injury were summarized in Table 1. The mechanisms of injury were classified into blade, saw, crush, and avulsion injuries.

**Table 1:** Demographic characteristics of 101 study participants by treatment group.

	Group I (n = 49)	Group II (n = 52)	*P* value
Age	40.1 ± 10.8	38.7 ± 9.7	0.19
Sex			
Male	43 (88)	47 (90)	0.67
Female	6 (12)	5 (10)	
Injury mechanism			
Blade	12 (24)	10 (19)	0.93
Saw	20 (41)	22 (42)	
Crush	10 (20)	12 (23)	
Avulsion	7 (15)	8 (16)	
Tamai level			
III	23 (47)	22 (42)	0.84
IV	21 (43)	23 (44)	
V	5 (10)	7 (14)	
Smoking			
Yes	20 (41)	26 (50)	0.35
No	29 (59)	26 (50)	

Group I: Nerve wrap; group II: control.

Figures are numbers (percentage).

### Nerve wrap

**Figure 1:**
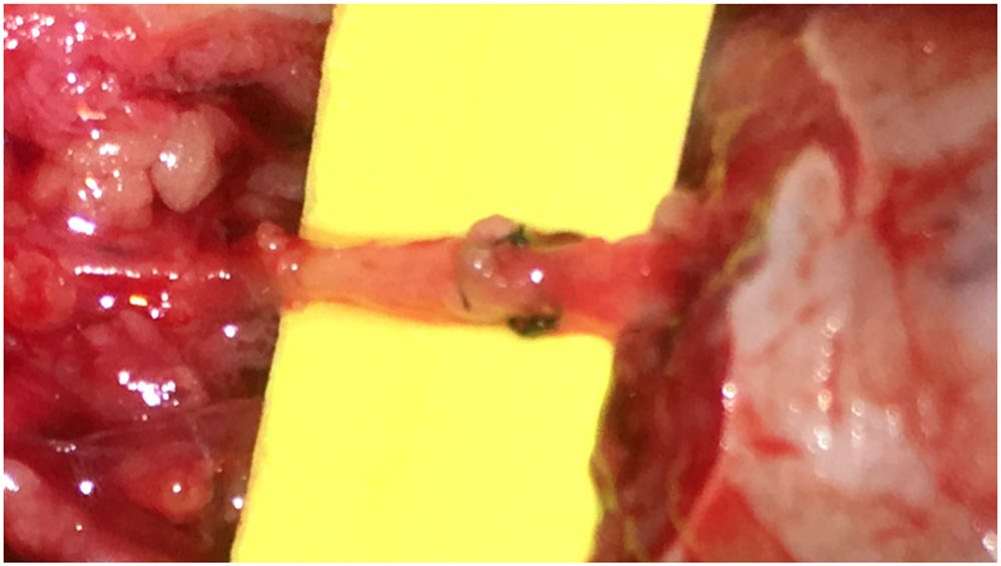
The nerves were anastomosed with epineurial fashion.

**Figure 2:**
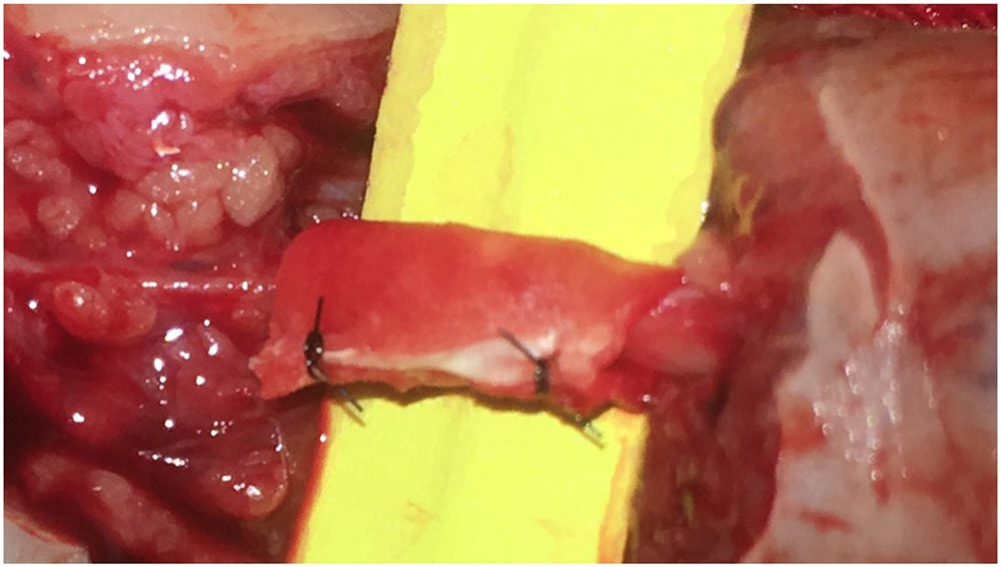
The repaired nerve was wrapped with the collagen conduit.

The nerves were first repaired using epineurial fashion technique with 10–0 nylon sutures under the operating microscope (Fig. 1). The current practices of nerve wrap after end-to-end and tension-free neurorrhaphy was empirical. Some surgeons in our hospital routinely conducted nerve wrap using a tendon-derived collagen conduit (Tianxinfu Medical Appliance, Beijing, China) while the other did not. For nerve wrap, the repaired nerve was first wrapped with the collagen conduit (with a gap) at the site of anastomosis and the gap was then closed with sutures (Fig. 2).

### Outcome assessment

Visual analog scale (VAS) score ranges from 0 (no pain) to 100 mm (worst pain possible). Because motion-evoked pain is frequently observed after nerve repair, similar to previous study^19^, we measured VAS score at rest and with exertion respectively according to the patient’s experience of average pain in the past week. The application of pain medication was also recorded. The static two-point discrimination (s2PD) were performed to evaluate sensory recovery. The s2PD test results was categorized as “Excellent” (≤6 mm), “Good” (7–15 mm), and “Poor” (>15 mm) respectively according to the Mackinnon classification^20^. VAS and s2PD tests were conducted at three and six months after surgery.

### Statistics

The significance of differences between groups in each variable was assessed using Pearson’s Chi-squared test or Student’s t test unless labeled otherwise. All data were presented as mean ± standard deviation.

## Results

The mean age of all patients was 39.4 ± 10.2 years. There were 90 amputated digits from men (89%) and 11 amputated digits from women (11%). Patients were divided into two groups based on whether received nerve wrap. The demographic and clinical characteristics were summarized in Table 1. All these variables had no significant differences between the two groups.

In group I (n = 49), the digital nerves were repaired with end-to-end and tension-free neurorrhaphy and then wrapped into a tendon-derived collagen nerve conduit. In group II (n = 52), the digital nerves were repaired with end-to-end and tension-free neurorrhaphy only. To evaluate the outcomes of sensory recovery, s2PD tests were conducted at three and six months after surgery. We did not observe any significant difference in sensory recovery after digit replantation between the two groups (Table 2). The VAS scores at rest and with exertion of group I were significantly reduced compared with those of group II at both three and six months after surgery (Table 3). Consistently, Tinel’s sign at the repair site was more frequently present in group II than group I.

**Table 2:** The s2PD of 101 study participants by treatment group.

	Group I (n = 49)	Group II (n = 52)	*P* value
Three months postoperatively			
Excellent	5 (10)	6 (12)	0.79
Good	25 (51)	23 (44)	
Poor	19 (39)	23 (44)	
Six months postoperatively			
Excellent	20 (41)	23 (44)	0.94
Good	25 (51)	25 (48)	
Poor	4 (8)	4 (8)	

Group I: Nerve wrap; group II: control.

**Table 3:** The VAS scores (mm, out of 100 mm) of 101 study participants by treatment group.

	Group I (n = 49)	Group II (n = 52)	*P* value
Three months postoperatively			
VAS score at rest	10.9 ± 8.3	14.7 ± 9.6	0.036
VAS score with exertion	28.9 ± 16.8	37.0 ± 18.7	0.025
Tinel’s sign (+)	29	42	0.029
Six months postoperatively			
VAS score at rest	7.7 ± 5.9	10.6 ± 6.9	0.027
VAS score with exertion	14.7 ± 14.9	23.7 ± 16.4	0.005
Tinel’s sign (+)	9	19	0.048

Group I: Nerve wrap; group II: control.

Traumatic neuroma was presented in three patients (all in group 2) by the end of follow up. The difference in the incidence of traumatic neuroma was insignificant between the two groups (*P* value = 0.24, Fisher’s exact test).

## Discussion

The conventional wisdom was that nerve warp could limit the regenerating axons in a proper orientation and thus bridge the gap between the two nerve stumps. For those with tension-free repair, nerve wrap was believed to be unnecessary^21^. However, recent progress has revealed that nerve wrap after end-to-end and tension-free neurorrhaphy could decrease perineural scar tissue formation and thus improve the prognosis in rats^16,17^. The clinical application of nerve wrap after end-to-end and tension-free neurorrhaphy is currently empirical and off-label, highlighting the need for clinical evidence. In this study, we show nerve wrap after end-to-end and tension-free neurorrhaphy could attenuate neuropathic pain after digit replantation but have no benefit for sensory recovery. Notably, digital nerves are sensory and it is still unclear whether nerve wrap could bring benefits for the injuries on motor and mixed nerve. Further studies are required to address this question.

There are various types of conduits differed with materials including collagen, biological and synthetic polymers^21^. In this study, we wrapped the anastomosed nerve with collagen conduits because the degradation of collagen could be completed within three months and delayed degradation leads to the formation of scar^22,23^. The currently available nerve conduits were generally designed for the treatment of nerve defect. Conceivably, these designs might not be optimal for nerve wrap after tension-free and end-to-end anastomosis. Novel designs might further increase the therapeutic benefits of nerve wrap.

The limitations of our study include a non-randomized design and a small sample size. Notably, all patients with traumatic neuroma belonged to group 2 and there is a clear tendency towards significance. A future study with larger sample size could address whether nerve wrap could reduce the incidence of traumatic neuroma.

## Conclusions

Nerve wrap into a collagen conduit after end-to-end and tension-free neurorrhaphy could attenuate neuropathic pain after digit replantation but show no benefit for sensory recovery.
